# The Pivotal Role of Consultant Physicians in the Early Screening, Diagnosis, and Management of Osteopenia and Osteoporosis: An Expert Opinion

**DOI:** 10.7759/cureus.116707

**Published:** 2026-09-22

**Authors:** Monica Mahajan, Manjunath Dinaker, Rajesh Uchil, Bijaya Kumar Mishra, Prashant Palkar, Krishna Shriram Dhanasekaran, Sucheta Mehta

**Affiliations:** 1 Department of Internal Medicine, Max Super Speciality Hospital, Saket, New Delhi, IND; 2 Department of Internal Medicine, GYD Diagnostics and Reference Laboratories (P) Ltd., Secunderabad, IND; 3 Department of Internal Medicine, Gleneagles Global Hospital, Mumbai, IND; 4 Department of Internal Medicine, Saraswat Clinic, Bhubaneswar, IND; 5 Medical Affairs, Cipla Ltd., Mumbai, IND

**Keywords:** bisphosphonates, bone health screening, consultant physicians, fragility fractures, osteopenia, osteoporosis

## Abstract

Osteoporosis and osteopenia are highly prevalent yet underdiagnosed metabolic bone conditions in India, contributing significantly to healthcare costs, morbidity, and mortality. While approximately one-fifth of Indian adults suffer from osteoporosis, nearly half present with osteopenia, frequently undiagnosed when an unexpected fragility fracture occurs. Consultant Physicians (CPs) serve as the primary point of contact for these patients and are uniquely positioned to spearhead early screening, diagnosis, and preventative interventions. Continued efforts to strengthen awareness among healthcare providers and patients, alongside broader access to screening and diagnostic tools, could facilitate earlier identification and timely intervention. This expert opinion paper emphasizes real-world clinical insights from senior CPs across India, integrating contemporary evidence to outline gaps in screening feasibility and primary care management. It underscores an urgent need to reposition metabolic bone health as a fundamental primary care priority and to empower CPs to lead targeted screening initiatives to facilitate early identification of individuals with osteopenia who have an elevated fracture risk and support timely interventions to prevent fragility fractures and further bone loss.

## Introduction and background

Osteoporosis is a systemic skeletal disorder characterized by low bone mass and deterioration of bone microarchitecture, resulting in increased bone fragility and risk for fractures [1]. In India, osteoporosis and osteopenia represent a major public health concern, driven by multiple factors including widespread vitamin D deficiency, inadequate nutritional intake, sedentary lifestyles, and the growing prevalence of chronic metabolic disorders such as diabetes mellitus [2,3]. In a large Indian observational study involving 31,238 adults, approximately 18% to 20% were reported to have osteoporosis and nearly half had osteopenia. These findings indicate a substantial burden among the evaluated population but still warrant nationally representative prevalence estimates [2]. Among postmenopausal women, the prevalence of osteoporosis may exceed 30% [2]. The disease burden is further influenced by the relatively earlier age of natural menopause in Indian women compared with Western populations, resulting in prolonged exposure to estrogen deficiency and accelerated bone loss [4,5].

Despite its high prevalence and associated risk of morbidity, osteoporosis remains underdiagnosed and undertreated in routine clinical practice [3]. Because bone loss occurs gradually and is typically asymptomatic until a fragility fracture occurs, opportunities for early diagnosis are often missed. Osteopenia, defined by a bone mineral density T-score between -1.0 and -2.5, represents an important stage in the spectrum of skeletal deterioration where timely identification and intervention may reduce the risk of progression to osteoporosis and future fractures [6]. Consultant Physicians (CPs), who often serve as the first point of contact for adults seeking medical care, are well positioned to identify individuals at risk, initiate appropriate screening, and implement preventive and therapeutic strategies for bone health [7,8].

Recent Indian guidelines have strengthened the evidence base for osteoporosis management; however, practical implementation within routine CP practice remains variable, particularly where access to dual-energy X-ray absorptiometry (DXA) and specialist services is limited. This article complements existing guidelines by translating contemporary evidence and regionally diverse clinical experience into a pragmatic, risk-based framework for early case-finding, diagnostic triage, comprehensive bone and muscle health assessment, and continuity of osteoporosis care in Indian clinical practice. Rather than functioning as a formal guideline, it aims to identify actionable opportunities for CPs and areas requiring further India-specific evidence.

## Review

Review purpose and methodology

This expert-opinion manuscript was developed through a targeted literature review and structured consultation with four senior CPs representing North, South, East, and West India, each with over 20 years of clinical experience.

Targeted Literature Review

A targeted search of PubMed/MEDLINE, Google Scholar, and relevant guideline and regulatory websites was conducted for English-language publications available up to January 2026. Search concepts included osteoporosis, osteopenia, fracture risk, Fracture Risk Assessment Tool (FRAX®) [9], DXA, quantitative ultrasound (QUS), sarcopenia, primary care, and India. Relevant guidelines, consensus statements, reviews, and clinical studies were selected based on recency, clinical relevance, methodological quality as reported in the original publications, and applicability to Indian practice. Consideration was given to recent Indian osteoporosis guidelines and contemporary international screening recommendations. As this was not a systematic review, Preferred Reporting Items for Systematic Reviews and Meta-Analyses (PRISMA)-based selection, formal risk-of-bias assessment, evidence grading, and quantitative synthesis were not performed.

Expert Consultations

The experts participated in four virtual meetings conducted between September 2025 and January 2026, each lasting approximately 60 minutes. A semi-structured discussion guide developed from the literature review was circulated during the consultation. Discussions addressed clinical awareness, risk identification, use of FRAX® [9] and DXA, diagnostic barriers, secondary causes, lifestyle and pharmacological management, sarcopenia, treatment monitoring, and referral considerations.

Synthesis of Recommendations

With participant agreement, discussions were recorded and synthesized by predefined domains. Differing opinions were reviewed against the available evidence, and the draft recommendations were revised and approved by all physician experts. No Delphi process, formal voting, or predefined consensus threshold was used; therefore, the recommendations represent evidence-informed expert opinion rather than formal consensus guidelines.

Governance and Author Independence

Authors affiliated with Cipla Medical Affairs supported literature review, collation, meeting coordination, documentation, and initial drafting. The physician experts independently assessed the clinical relevance of the evidence and retained authority to modify or reject any recommendations, including those concerning drug selection, dosing, and treatment positioning. Medications and class descriptions were intended to reflect approved indications and published guidance. No specific proprietary brand is recommended in the manuscript. No recommendation was included without expert review and approval. All authors reviewed and approved the final manuscript.

Expert deliberations and clinical barriers

Consultant Physicians as the Primary Gatekeepers for Bone Health

The nationwide POSEIDON one-day point-prevalence study documented symptoms and medical conditions among 204,912 patients attending primary healthcare practitioners across India, illustrating the broad clinical interface available to primary-care physicians for identifying comorbid and preventive health needs [8]. Yet, despite this high baseline exposure, initially silent skeletal conditions like osteopenia and osteoporosis are consistently under-recognized compared to respiratory, circulatory, or gastrointestinal complaints, even among high-risk individuals aged over 50 years [3,8].

Without pain or visible symptoms, patients lack a physical trigger to seek care. Osteoporosis is frequently asymptomatic until a fragility fracture occurs. The expert panel observed that bone health is therefore rarely the primary reason for consultation, and opportunities for risk assessment may be missed when attention is focused on symptomatic comorbidities. Diffuse symptoms like chronic low back pain, localized hip discomfort, or generalized bone pain, frequently experienced by postmenopausal women, are routinely self-dismissed as normal signs of aging or physical stress. They should not be considered diagnostic of osteoporosis, but should prompt bone health evaluation.

CPs routinely co-manage chronic conditions like diabetes, thyroid imbalances, and lifestyle-related diseases. Notably, a large proportion of patients do not directly consult bone health specialists, being asymptomatic at the initial stages. This places CPs in a pivotal position towards screening and managing bone health at early stages. They can identify clinical risk factors, evaluate potentially reversible secondary causes, and arrange appropriate testing, treatment, or specialist referral before a fragility fracture occurs.

Challenges in Osteoporosis Recognition and Prioritization

A qualitative study by Salminen H et al. highlighted that primary care physicians (PCPs) frequently perceive osteoporosis as a lower clinical priority, driven in part by the asymptomatic nature of the disease, the perceived complexity of long-term metabolic bone management, and the lack of immediate benefit with anti-osteoporotic medications [10]. Competing, chronic medical disorders with acute episodic symptoms (such as hypertension and diabetes, etc.) are regularly prioritized during consultation by both physicians and patients. Findings by Thakur P et al. demonstrated significant knowledge gaps among practicing medical professionals regarding standard osteoporosis diagnostic workflows and structured treatment frameworks [11].

Thus, lower prioritization eventually contributes to delay in timely risk identification and prevention, allowing silent bone loss to advance into fragility fractures. These challenges are influenced by limited emphasis on metabolic bone health during medical training, variable adoption of the validated FRAX® tool [9], and unequal access to diagnostic modalities, including DXA, particularly in non-metropolitan settings.

The Suboptimal Triage of Osteopenia: The Missed Opportunity for Prevention

As per the World Health Organization (WHO), osteopenia is defined by bone densitometry as a T-score between -1.0 and -2.5 [1]. Although osteoporosis is associated with a higher individual fracture risk, osteopenia contributes substantially to the overall fracture burden because of its far greater prevalence in the population. Studies have shown that the proportion of fractures occurring in individuals with osteopenia (52%) exceeds that occurring in individuals with osteoporosis (18%), highlighting the importance of early identification and intervention in this seemingly lower-risk group [6]. Despite this clinical reality, osteopenia is widely overlooked or viewed as a benign condition in routine primary care practice [12].

Many practitioners may still exclusively associate metabolic bone loss only with advanced age or postmenopausal status, leaving younger, at-risk individuals entirely unscreened. This clinical blind spot creates a missed opportunity, where patients progressively fall through the cracks of the healthcare system due to systemic screening drop-offs, cost barriers, and a low utilization of primary risk tools [6,13].

The Silent Burden of Osteoporosis: Consultant Physicians Must Lead Early

While osteopenia represents an important opportunity for early intervention, osteoporosis constitutes the stage at which skeletal fragility becomes clinically significant. Fracture risk increases as bone mineral density (BMD) declines and is further influenced by age, previous fracture, falls, comorbidities, and other clinical risk factors. Defined by a T-score ≤ -2.5, osteoporosis is frequently diagnosed only after the occurrence of a fragility fracture, thereby reflecting a failure of timely identification and preventive care [1,6]. Vertebral, hip, wrist, and proximal humerus fractures are often the first manifestations of underlying osteoporosis in routine clinical practice. Such fractures are associated with substantial morbidity, loss of independence, reduced quality of life, healthcare expenditure, and increased mortality, particularly among older adults [2,14,15].

Based on their collective clinical experience, the expert panel considered that CPs should be well positioned to identify individuals with established risk factors, evaluate secondary causes, initiate guideline-consistent management when appropriate, and arrange specialist referral for complex or very-high-risk patients. Given that osteoporosis frequently coexists with chronic disorders such as diabetes mellitus, chronic kidney disease, thyroid dysfunction, inflammatory diseases, and prolonged glucocorticoid exposure, CPs are uniquely positioned to integrate bone health assessment into routine chronic disease management. Early diagnosis and treatment of osteoporosis can significantly reduce future fracture risk and should be regarded as an essential component of preventive healthcare rather than a reactive response to fracture events.

Risk-based screening and stepwise diagnostic approach

To overcome the limitations of age-based screening alone, a risk-based case-finding strategy for the early detection of both osteopenia and osteoporosis is strongly recommended. Given the limited availability and affordability of DXA in many parts of India, CPs should adopt a pragmatic, stepwise diagnostic approach that combines clinical risk assessment, fracture risk estimation, and selective use of imaging modalities (Table 1).

**Table 1:** Expert-Opinion Practical Screening and Diagnostic Framework for Osteopenia and Osteoporosis in Routine Clinical Practice FRAX: Fracture Risk Assessment Tool [9], QUS: Quantitative Ultrasound, DXA: Dual-Energy X-ray Absorptiometry, BMD: Bone Mineral Density

Step	Approach	Assessment	Practical Objective
1.	Risk Profiling	Identify at-risk individuals based on age, menopause status, prior fractures, comorbidities, medications, falls, lifestyle factors, and family history	Early recognition of patients with potential bone loss
2.	Clinical Fracture Risk Assessment	Perform FRAX® assessment, with or without BMD, to estimate 10-year fracture risk [9]	Identify osteopenic patients requiring further evaluation or treatment
3.	Initial Screening	Utilize QUS and/or spine X-ray radiographs where DXA is inaccessible	Detect low bone mass and occult vertebral fractures
4.	Laboratory Evaluation	Assess calcium, phosphate, alkaline phosphatase, vitamin D status, renal function, thyroid function, and other relevant investigations	Identify secondary causes of osteoporosis
5.	Targeted DXA Assessment	Perform central DXA of the lumbar spine and hip in individuals with high-risk	Confirm diagnosis of osteopenia or osteoporosis and guide therapy
6.	Risk Stratification & Management Planning	Categorize patients as normal BMD, osteopenia, osteoporosis, or severe osteoporosis and initiate appropriate interventions	Align treatment intensity with fracture risk
7.	Follow-up & Monitoring	Reassess adherence, falls risk, interval fractures, and repeat BMD check	Monitor disease progression and treatment response

Stepwise Diagnostic Approach

Universal clinical risk profiling:** **As bone loss starts as early as 40-45 years of age in Indian women, using a structured risk-factor assessment (Table 2), irrespective of symptoms, may be necessary [14].

**Table 2:** High-Risk Clinical Profiles Requiring Screening and Diagnostic Evaluation BMI: Body Mass Index

Risk Category	High-Risk Individuals
Age & Hormonal Factors	Women ≥45 years, postmenopausal women, perimenopausal women, and men ≥50 years
Prior Skeletal Events	History of low-trauma or fragility fractures, documented height loss, or suspected vertebral compression fractures
High-Risk Medications	Chronic glucocorticoids, antiepileptic drugs, antiretroviral therapy, heparin, methotrexate, and prolonged proton pump inhibitor therapy
Comorbid Conditions	Type 2 diabetes mellitus, juvenile-onset diabetes, chronic kidney disease, chronic liver disease, rheumatoid arthritis, ankylosing spondylitis, inflammatory bowel disease (IBD), thyroid and parathyroid disorders
Lifestyle & Nutritional Factors	Low BMI, sedentary lifestyle, inadequate calcium or protein intake, smoking, excessive alcohol consumption, recurrent falls, frailty, and prolonged immobilization

Non-BMD fracture risk estimation: Incorporate FRAX® during routine consultations to estimate 10-year major osteoporotic and hip fracture risk, even in the absence of DXA data [9,16-19].

Preliminary structural triage: In settings where central DXA is not immediately available, calcaneal QUS may contribute to preliminary fracture-risk assessment, while lateral spine radiography may identify suspected vertebral compression fractures in appropriately selected patients. Neither QUS nor conventional radiography should be used as a substitute for central DXA to diagnose osteoporosis. Individuals with abnormal QUS findings, suspected vertebral fracture, or high clinical fracture risk should undergo central DXA and/or specialist evaluation when feasible [7,13].

Targeted DXA confirmation:* *Reserve DXA for patients identified as high-risk through clinical assessment, FRAX®, or preliminary imaging findings, thereby optimizing resource utilization [7,9,14,16].

Holistic management strategies for osteopenia and osteoporosis

Management must look beyond simple pharmacotherapy to combine lifestyle corrections with medication adherence [14,20].

Lifestyle, Nutrition, and Sarcopenia Assessment

Lifestyle and nutritional interventions are foundational across the osteopenia-osteoporosis spectrum and should be implemented irrespective of pharmacologic therapy. As highlighted in the Indian Orthopaedics Association (IOA) 2025 guidelines, optimizing bone and muscle health is essential for reducing fracture risk and preserving function [14]. In India, inadequate protein and calcium intake, vitamin D deficiency, and sedentary lifestyles contribute significantly to bone loss, particularly among postmenopausal women and older adults. The coexistence of osteoporosis and sarcopenia further increases the risks of frailty, falls, and fractures, underscoring the importance of routinely assessing both bone and muscle health in clinical practice [14]. A multi-dimensional preventive strategy is recommended as follows.

Optimizing nutrition intake: Ensure adequate daily calcium and vitamin D through diet and supplementation, and individualized protein intake to support both skeletal and muscle health with appropriate supplementation. Particular attention should be paid to elderly individuals, postmenopausal women, and patients with chronic illnesses or undernutrition [14,20].

Physical activity and muscle strengthening: Encourage regular weight-bearing exercises, resistance training, balance training, and muscle-strengthening activities. These interventions improve BMD, preserve muscle mass, enhance physical performance, and reduce falls risk [15].

Osteosarcopenia assessment:* *Evaluate for muscle weakness, reduced physical performance, recurrent falls, frailty, and loss of functional independence, particularly in older adults. Early identification of osteosarcopenia allows timely implementation of nutritional and exercise-based interventions [15].

Behavioral modification and fall prevention: Promote smoking cessation, moderation of alcohol consumption, maintenance of healthy body weight, vision and gait assessment, medication review, and home safety measures to minimize fall risk. Fall prevention should be considered an integral component of fracture prevention [14].

Standardized Pharmacological Management

Pharmacological management of metabolic bone disease should follow a risk-based approach aimed at preventing fragility fractures and reducing progression from osteopenia to osteoporosis. CPs play a crucial role in identifying high-risk individuals and initiating timely therapy. Available treatment options include major pharmacological classes such as bisphosphonates, calcitonin receptor agonists, RANKL inhibitors, and anabolic agents, with treatment selection guided by fracture risk, disease severity, patient characteristics, and approved indications. An overview of representative pharmacological classes, agents, approved indications, and dosing regimens in India is provided (Table 3). This overview is intended as a drug class-based clinical reference relevant to CPs in their practice and should not be considered an exhaustive list of all available products, formulations, or brands.

**Table 3:** Pharmacological Management of Osteopenia and Osteoporosis: Approved Therapies and Clinical Indications PO: Oral; IV: Intravenous; SC: Subcutaneous; RANKL: Receptor Activator of Nuclear Factor Kappa-B Ligand; PTH: Parathyroid Hormone; ROA: Route of Administration Note: Doses and indications should be verified against the current Indian prescribing information for the specific product and formulation before use. Approved indications, contraindications, renal restrictions, and duration limits may differ between agents and countries other than India. This table is a non-exhaustive class-based summary and does not replace the applicable prescribing information or clinical judgment. Intranasal calcitonin is not considered first-line therapy for osteoporosis. Clinicians should consult the current Indian prescribing information for approved indications, duration, contraindications, and safety warnings.

Class (Mechanism of Action)	Drug	Dose, ROA & Frequency	Approved Indications in India
Bisphosphonate (anti-resorptive)	Low dose Alendronate	35 mg PO once weekly	Prevention of osteoporosis in post-menopausal women
	Alendronate	70 mg PO once weekly	Treatment of osteoporosis in post-menopausal, male and Glucocorticoid-induced osteoporosis
	Risedronate	35 mg weekly / 150 mg monthly	Prevention and treatment of post-menopausal osteoporosis treatment of male and glucocorticoid-induced osteoporosis
	Ibandronate	150mg PO monthly	For the treatment and prevention of postmenopausal osteoporosis
	Zoledronic Acid	5mg/100ml solution for infusion once yearly	For the treatment of post-menopausal & male osteoporosis at increased risk of fracture, including those with a recent low-trauma hip fracture
Calcitonin receptor agonist (anti-resorptive & analgesic)	Intranasal Salmon Calcitonin	200 IU nasal spray	Treatment of post-menopausal osteoporosis, bone pain associated with osteolysis and/or osteopenia and Paget's disease.
RANKL Inhibitor (anti-resorptive)	Denosumab	60 mg SC every 6 months	Treatment of postmenopausal, male and glucocorticoid-induced osteoporosis at high risk of fracture.
PTH Analog (Anabolic)	Teriparatide	20 μg SC daily (maximum 24 months)	Treatment of severe osteoporosis with high risk for fracture

Osteopenia (T-score -1.0 to -2.5): Osteopenia represents a critical window for prevention. Lifestyle modification, adequate calcium and vitamin D intake, optimized protein nutrition, and regular weight-bearing exercise remain the cornerstone of management. Along with BMD T-score, pharmacotherapy can be considered in osteopenic individuals with clinical risk factors (Table 2) as per the country-specific guidelines. Among available therapies, low-dose alendronate (35 mg once weekly) is particularly relevant as it is approved for the prevention of postmenopausal osteoporosis as a fracture risk-based pharmacotherapy. 

Established osteoporosis (T-score ≤ -2.5 with or without fragility fracture): Most patients with confirmed osteoporosis should be evaluated for pharmacological treatment, with the final decision individualized according to fracture risk, age, previous fractures, secondary causes, life expectancy, renal function, contraindications, patient preferences, and the reliability of the BMD assessment.

Oral bisphosphonates, particularly alendronate and risedronate, remain the preferred first-line agents due to their proven efficacy, cost-effectiveness, and fracture risk reduction. Intravenous zoledronic acid is a suitable alternative in selected patients, particularly when adherence or gastrointestinal tolerability is a concern. For patients at high or very high fracture risk, denosumab offers a potent antiresorptive option and is particularly useful in patients intolerant to bisphosphonates or with specific clinical considerations. However, discontinuation requires switching to sequential antiresorptive therapy to prevent rebound bone loss.

Intranasal salmon calcitonin, 200 IU once daily, can be added for reduction when suitable alternatives are not appropriate. Its role in osteoporosis is limited because of modest antifracture evidence and a reported malignancy signal with long-term calcitonin exposure. Continued need should be periodically reassessed.

Severe osteoporosis:* *Severe osteoporosis characterized by very poor BMD, or persistent high fracture risk, recurrent or multiple fragility fractures, suspected secondary osteoporosis, major renal impairment, uncertainty regarding diagnosis, treatment failure, intolerance or contraindication to available therapies, and patients being considered for anabolic or other advanced treatment strategies. Specialist referral can be considered in these cases. Teriparatide as an anabolic therapy may be considered to stimulate new bone formation. Following completion of teriparatide, an antiresorptive agent should be initiated to maintain treatment gains.

Overall, the treatment decision should be aligned with fracture risk, with osteopenia viewed as an opportunity for prevention and osteoporosis as a condition requiring structured long-term management to reduce future fractures and preserve functional independence.

To safely guide long-term therapeutic success, CPs should be familiar with two critical needs as follows [7].

Individualized Reassessment and Bisphosphonate Holiday

To minimize the rare, long-term risks of atypical femoral fractures or osteonecrosis of the jaw, oral alendronate therapy should be re-evaluated after three to five years of continuous and compliant use [14]. In patients with stable BMD, low continuing fracture risk, and no recent history of skeletal fractures, a structured drug holiday may be considered [14]. This decision should be individualized according to the patient’s ongoing fracture risk, prior fractures, BMD, glucocorticoid exposure, and other clinical risk factors; treatment should be reconsidered if a new fracture occurs, BMD declines meaningfully, or fracture risk increases. Bone health should continue to be monitored during this temporary treatment interruption.

Transition Mechanics After Denosumab Discontinuation

Planned treatment following denosumab discontinuation: Denosumab should not be delayed or discontinued without a planned, guideline-consistent transition to another antiresorptive therapy because bone turnover can increase rapidly after its effects diminish, with rebound BMD loss and increased risk of vertebral fractures [14,21]. Sequential therapy is generally initiated around the time the next denosumab dose would have been due, but the choice and timing of treatment should be individualized according to fracture risk, duration of denosumab exposure, renal function, prior therapy, BMD, and, where available, bone-turnover markers. Specialist input should be considered for high-risk or complex patients.

Discussion

Osteopenia and osteoporosis remain highly prevalent yet underrecognized conditions in India, frequently remaining undiagnosed until the occurrence of a fragility fracture. This underscores the need to shift from a reactive fracture-focused model toward proactive risk identification and prevention. Given their role as the first point of healthcare contact for many patients, CPs are well positioned to facilitate early detection, risk stratification, and timely intervention for individuals at risk of skeletal fragility [2,3,8].

A key recommendation was that osteopenia should be viewed as an important opportunity for preventive intervention rather than a benign precursor state. Considering the limited accessibility of DXA in several parts of India, a pragmatic risk-based strategy incorporating clinical risk assessment, FRAX® evaluation, and selective use of diagnostic investigations may improve the identification of individuals at increased fracture risk while optimizing resource utilization [7,9,13,17-19].

The experts further emphasized that effective bone health management extends beyond pharmacological therapy alone. Adequate nutrition, calcium and vitamin D optimization, regular weight-bearing exercise, fall-prevention measures, and assessment of coexisting sarcopenia are integral components of comprehensive care. Addressing both skeletal and muscle health may help reduce fracture risk, preserve functional independence, and improve long-term outcomes among older adults [14,20].

Collectively, these observations support the need for greater awareness, structured screening pathways, and wider adoption of evidence-based bone health practices in primary care. Strengthening physician education, improving access to diagnostic tools, and generating India-specific clinical data may help bridge existing practice gaps and enhance the early detection and management of osteopenia and osteoporosis in routine clinical practice (Table 4) [2,11,14].

**Table 4:** Summary of Expert-Opinion Recommendations for Early Screening, Diagnosis, and Management of Osteopenia and Osteoporosis in India BMI: Body Mass Index; FRAX: Fracture Risk Assessment Tool [9]; QUS: Quantitative Ultrasound; DXA: Dual-Energy X-ray Absorptiometry

Approach	Practical Recommendation
Crucial Role of Consultant Physicians	Integrate bone health assessment into routine evaluation of middle-aged and older adults, particularly those with chronic metabolic and endocrine disorders, and lead early risk stratification, screening, and management of osteopenia and osteoporosis.
Early Detection	Routinely screen women ≥45 years, postmenopausal women, men ≥50 years, and younger individuals with risk factors such as prior fragility fractures, chronic glucocorticoid use, diabetes, chronic kidney disease, low BMI, family history of osteoporosis, or recurrent falls.
Risk-Based Assessment	Use structured risk-factor assessment and FRAX^®^ scoring to identify individuals at increased fracture risk and guide decisions regarding further evaluation and treatment.
Diagnostic Strategy	Adopt a stepwise diagnostic approach using clinical assessment, FRAX^®^, QUS and/or spine radiographs where appropriate, with targeted DXA testing for high-risk or clinically suspicious individuals.
Osteopenia Management	Recognize osteopenia as an important opportunity for fracture prevention and initiate lifestyle interventions and pharmacotherapy in appropriate high-risk patients.
Musculoskeletal Health Optimization	Routinely assess sarcopenia, frailty, falls risk, and functional status, and incorporate nutrition optimization, adequate protein intake, resistance training, and balance exercises alongside bone-directed therapy.
Treatment	Individualize treatment according to fracture risk and disease severity, while ensuring adherence to recommended bisphosphonate holiday protocols and appropriate sequential antiresorptive therapy following denosumab discontinuation.
Patient Education	Counsel patients on bone health, calcium and vitamin D intake, physical activity, smoking cessation, fall prevention, and treatment adherence to improve long-term outcomes.
Physician Capacity Building	Strengthen Continuing Medical Education (CME) programs and provide practical clinical algorithms to improve awareness, confidence, and consistency in osteoporosis care.
Clinical Guidelines & Standardization	The Association of Physicians of India (API) should consider developing practical, India-specific guidance for Consultant Physicians on the screening, diagnosis, risk stratification, and management of osteopenia, osteoporosis, and sarcopenia. Such guidance could provide standardized screening criteria, FRAX^® ^tool-based risk assessment, treatment thresholds, monitoring recommendations, and referral algorithms to promote uniformity of care across primary care settings.
Healthcare Infrastructure	Improve access to DXA services, bone health clinics, community screening initiatives, and fall-prevention programs, particularly in underserved settings.
Evidence Generation	Promote India-specific epidemiological studies, registries, and real-world evidence generation on osteopenia, osteoporosis, sarcopenia, diabetic osteopathy, and fracture outcomes to inform clinical practice and policy.
National Health Policy Integration	Recognize osteopenia, osteoporosis, and sarcopenia as important public health priorities and integrate bone and muscle health assessment into healthy ageing and non-communicable disease (NCD) frameworks. Strengthening epidemiological surveillance and evidence generation can support policy development, resource allocation, standardized care pathways, and implementation of scalable fracture-prevention strategies.

Limitations

This expert opinion is based on a targeted review of the literature and structured expert consultations and should therefore be interpreted as expert guidance rather than a formal consensus guideline. A formal Delphi process, quantitative voting, predefined consensus thresholds, or systematic evidence grading was not employed. Additionally, the recommendations are derived from discussions among four experienced CPs and may not fully capture all regional and practice-level variations across India. Nonetheless, the inclusion of experts from diverse geographic regions and the integration of contemporary published evidence provide practical insights relevant to routine clinical practice. Further real-world studies and broader multi-stakeholder consensus initiatives are needed to validate and refine these recommendations.

## Conclusions

Osteopenia and osteoporosis represent a growing yet largely underrecognized public health challenge in India. Given their unique position at the frontline of healthcare, CPs are ideally placed to identify at-risk individuals, initiate timely screening, implement evidence-based interventions, and prevent progression to fragility fractures. A pragmatic, fracture risk assessment using FRAX^® ^tool, BMD evaluation through DXA followed by lifestyle optimization and individualized pharmacotherapy may support earlier risk identification and appropriate management. Increasing the awareness of patients and health care professionals (HCPs), improving access to diagnostic services by integrating bone and muscle health into routine clinical practice may help reduce missed opportunities for fracture prevention. The effectiveness and feasibility of the proposed approach should be evaluated through nationwide implementation, epidemiological and real-world outcome studies.
